# Doppler ultrasound in the measurement of pulse wave velocity: agreement with the Complior method

**DOI:** 10.1186/1476-7120-9-13

**Published:** 2011-04-15

**Authors:** Jordi Calabia, Pere Torguet, Maria Garcia, Isabel Garcia, Nadia Martin, Bernat Guasch, Diana Faur, Martí Vallés

**Affiliations:** 1 Department of Nephrology, Hospital Universitari Dr. J.Trueta de Girona, Av. de França s/n, Girona, Spain; 2 School of Medicine, University of Girona, Girona, Spain; 3 Research support Unit, IDIAP Jordi Gol, Girona, Spain

## Abstract

Aortic stiffness is an independent predictor factor for cardiovascular risk. Different methods for determining pulse wave velocity (PWV) are used, among which the most common are mechanical methods such as SphygmoCor or Complior, which require specific devices and are limited by technical difficulty in obtaining measurements. Doppler guided by 2D ultrasound is a good alternative to these methods. We studied 40 patients (29 male, aged 21 to 82 years) comparing the Complior method with Doppler. Agreement of both devices was high (R = 0.91, 0.84-0.95, 95% CI). The reproducibility analysis revealed no intra-nor interobserver differences. Based on these results, we conclude that Doppler ultrasound is a reliable and reproducible alternative to other established methods for the measurement of aortic PWV.

## Introduction

Large arteries are not simple tube conduction structures. They moderate systolic pressure increases and maintain sufficient diastolic level to guarantee myocardial perfusion. With the identification of new diseases and risk factors, it has been seen that these arteries lose their natural elasticity leading to high systolic and low diastolic blood pressure levels, which determine high pulse pressure.

Based on these premises, arterial stiffness is now considered an increasingly important biomarker in the evaluation of cardiovascular risk and the detection of incipient vascular disease. Several studies have shown that this parameter is an independent predictor of cardiovascular mortality in the elderly, hypertensive, diabetics, and patients with chronic renal failure as well as in the general population [1-4]. The guidelines of the European Societies of Hypertension and Cardiology (2007-2009) have postulated arterial stiffness assessment, measurement of the carotid plaque and ankle/brachial index as markers of vascular status. Any alteration of these measurements may define a state of vasculopathy that significantly increases the evaluation of risk [5].

Among the different methods of evaluating arterial stiffness, the most widely used in the literature is aortic pulse wave (PWV), specifically in the area running from the aortic arch or common carotid artery to the common femoral artery. Typically, the pulse wave is detected by pressure transducers or arterial tonometry.

**Figure 1:**
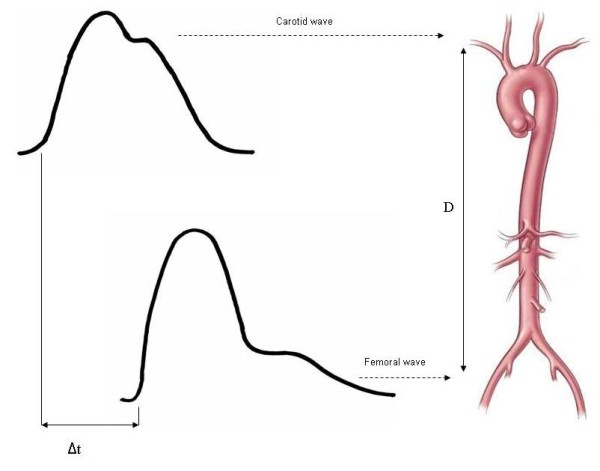
**Pulse wave velocity determination**. Transit time is estimated by the foot-to-foot method. The foot of the wave is defined at the end of diastole, when the steep rise of the waveform begins. The transit time is the time of travel of the foot of the wave over a known distance.

The measurement of carotid-femoral PWV (Figure 1) is made by dividing the distance (from the carotid point to the femoral point) by the so-called transit time (the time of travel of the foot of the wave over the distance). Hence, PWV = D (meters)/Dt (seconds) [6,7].

Whereas the distance is a fixed parameter, the transit time has a certain variability, depending on factors such as cardiac conduction and rhythm. Given this situation, most methods take the average of several measurements.

These methods are highly reliable but have the disadvantages of requiring specific devices and software and of sometimes being impossible to perform accurately due to the difficulty in recording good pulse waves. Furthermore, the time required for the exploration is not negligible.

These disadvantages are overcome if we take the carotid-femoral PWV measurement by ultrasound, making the assumption that real pulse wave corresponds to the flow wave of spectral Doppler. In fact, this method has been used in population-based studies such as the ABC study [8-13]. On this basis, we designed a comparative study to assess whether PWV measured by mechanical pressure and PWV estimated by ultrasound are similar, and reliable in the measurement of arterial stiffness.

## Material and methods

The subjects studied were patients from the Cardiovascular Risk and Hypertension Unit of the Dr. Josep Trueta University Hospital. Inclusion criteria were patients with essential hypertension, diabetes mellitus or chronic kidney disease and indications to testing were the evaluation of specific cardiovascular risk. Exclusion criteria were atrial fibrillation, severe cardiac valve disease and the presence of a prosthetic aorta. Both PWVc-f by the Complior^® ^method and Doppler ultrasound measurements were performed on all patients by two investigators. Other clinical variables such as blood pressure and heart rate were also recorded. The study was approved by the Hospital Dr. Josep Trueta Ethics Committee.

### PWV measured by mechanotransducers

The Complior^® ^System (Artech Medical, Pantin, France) was used as the method of reference to determine PWVc-f, which was automatically calculated as the average of 8-10 transit times and the distance, measured from the sternal notch to the femoral artery at the groin. The test was performed in supine position, placing sensors at the carotid and femoral pulses (at the area of maximum heart rate by palpation). The result was the average of two or three speed measurements.

### PWV measured by Doppler

**Figure 2:**
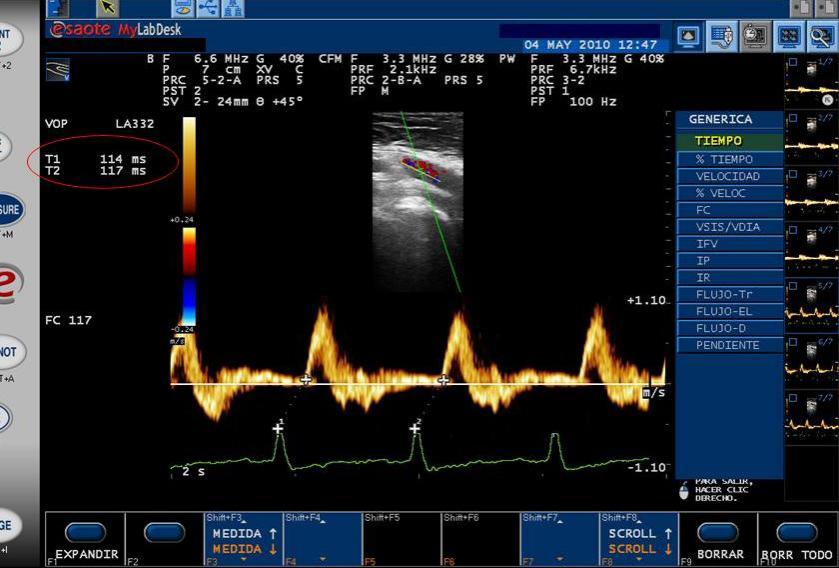
**Time measurement in femoral arthery gatting with ECG in two heart rates**.

Although it is not possible to analyze the carotid and femoral waves simultaneously, they can be normalized separately with the electrocardiogram (ECG) (gatting). We used a pulsed Doppler ultrasound with a Linear Array (6.6 MHZ) probe, synchronized with ECG and a two-second minimum sliding window (MyLab25, Esaote, Florence, Italy). The examination began with the patient in a supine position after locating the carotid artery with B-mode at the supraclavicular level (1-2 cm of the bifurcation). We then identified the wave Doppler flow simultaneously with ECG. The process was repeated on the common femoral artery in the groin. We performed three recordings of the carotid artery and three recordings of the femoral artery in the groin. Each recording involved two or three cardiac cycles. To find the transit time (TT), we measured the time from the R wave of QRS to the foot of the waveform using digital calipers (Figure 2). Six heart rate measurements were taken and the average was calculated. To determine the velocity, we used the same distance as the Complior^® ^system.

To check reproducibility two blinded observers separately measured the PWV using the Doppler images of 10 consecutive patients. One observer later repeated the measurements twice at different times.

## Sample size

The available sample size (n = 40) provided power >90% at 1% significance level in order to contrast the null hypothesis that the ICC equals 0.7 and the alternative hypothesis that the ICC equals 0.9 (two-sided). StudySize 2.0 Trial software was used for sample size calculations.

## Statistical analysis

Continuous variables were described with mean and standard deviation and percentages were used to describe qualitative variables. Agreement between PWV measured by Doppler and Complior as well as the intra-and inteobserver agreement of the new technique was calculated based on intraclass correlation coefficient (ICC). Bland-Altmann plots were used to determine precision and bias between methods. The analysis is based on the examination of two graphs: first, the identity plot (a scatterplot of the two measurements along with the line y = x); second, the plot of the difference between methods against the gold standard (Complior).

## Results

A heterogeneous group of 47 patients aged from 21 to 82 years were studied in 2009 and 2010. Seven patients were excluded, three with atrial fibrillation, three for failure to perform the Complior technique (inability to find a correct carotid pulse wave) and one due to the presence of aortic prostheses.

The sample characteristics are detailed in Table 1. PWV Complior (9.81 ± 2.76) ranged from 4.13 m/s to 19.9 m/s, similar to the PWV Doppler (9.95 ± 3.13) from 3.96 m/s to 20.2 m/s. The mean difference between the two measures was virtually the same 0.13 (0.19). The two methods showed very good agreement (R = 0.91, 0.84-0.95; CI 95%). The analysis of reproducibility also showed very good agreement (Table 2)

**Table 1:** Subject characteristics (n = 40)

Variable	Mean or number of patients (s.d. or %)
Gender (F/M)	11 (27.5%)/29(72.5%)
Age (years)	59 (15.8)
Diabetes Mellitus	11 (27.5%)
Cronic kidney failiure (FG < 60 ml/min)	21 (52.5%)
Sistolic BP (mmHg)	131 (16.4)
Diastolic BP (mmHg)	74 (10.5)
Path length (cm)	54.3 (4.5)

**Table 2:** Agreement between methods and intraobserver and interobsever reproductibility

	ICC*	Mean differences	Standard error of mean difference
**Between methods**	0.91 (0.84-0.95)	0.13	0.19
**Intraobserver**	0.98 (0.92-0.99)	0.32	0.29
**Interobserver**	0.97 (0.89-0.99)	-0.18	0.38

**Figure 3:**
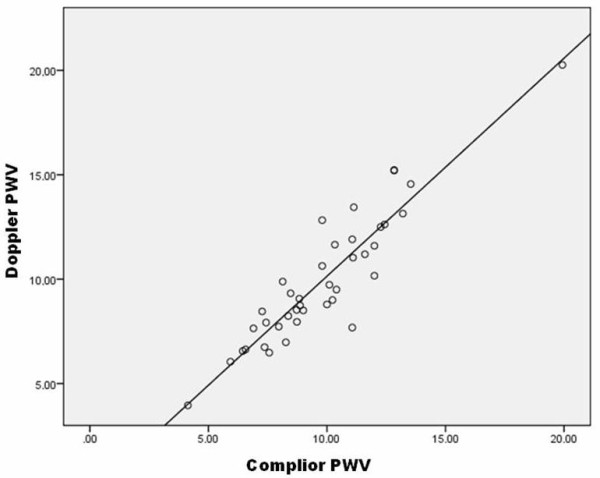
**Identity plot. Comparison of Doppler and Complior PWV measurement methods**.

**Figure 4:**
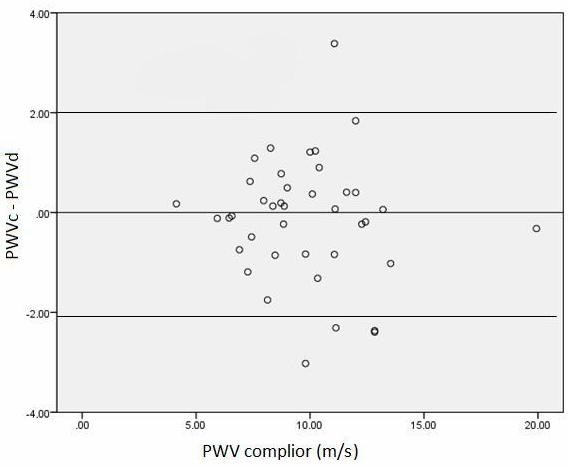
**Bland and Altman plot of Complior vs. Doppler PWV with mechanical method as reference**. The intervals of two standard deviations are considered as the concordance limits between the two measurements, accounting for 95% of the observed differences.

The identity plot in Figure 3 showed basic agreement between the two methods since the scatterplot lined up closely to the line y = x. Moreover, the Bland-Altman plot (Figure 4) indicated that the 95% limits of agreement ranged from -2 to +2. Hence, the two methods provided similar measures as the level of disagreement did not include clinically important discrepancies.

## Discussion

Age, atherosclerosis and the presence of certain diseases are known to decrease arterial elasticity. As can be seen in Table 3, there are many methods to estimate arterial stiffness, which has now taken on great importance in assessing cardiovascular risk. Non-invasive methods are based on local, regional or systemic measures. Direct measurement of local stiffness is usually made by echotracking systems, which measure in situ the differences of arterial diameter over the wave flow.

**Table 3:** Devices and methods used to determine arterial stiffness

Non-invasive	**Regional stiffness**:	Mechanotransducer
		
	Pulse wave velocity	Tonometer
		
		Echotracking
		
		Doppler
	
	Local stiffness	Echotracking
		
		Magnetic resonance
	
	Systemic stiffness	Waveform shape analysis
Invasive	**Aortic angiography**	

The most commonly used regional method is aortic pulse wave velocity, which requires two variables: the distance between two points in the artery and the time taken by the pulse to cover that distance. Carotid-femoral PWV is a simple, non-invasive, robust and reproducible method that is regarded as the gold standard for measuring arterial stiffness since epidemiological studies have found it to be an independent predictor of cardiovascular events.

The Complior System^®^, which uses two mechanotransducers applied to the skin and measures real-time pulse waves at carotid and femoral points, is used in most of these studies. Another widely used system is SphygmoCor^®^, which uses an applanation tonometer [6-16]. This device can also calculate the central pressure and augmentation index.

Complior records both waves simultaneously, whereas SphygmoCor records consecutively using ECG. In this case, changes in heart rate between two recordings may determine a variation in transit time.

These mechanical methods have the disadvantages that there is a prolonged learning period in order to become an experienced observer and that the devices used lack versatility. Furthermore, the technical difficulty in obtaining measurements and anatomical limitations of some patients make it necessary to find faster and more versatile methods for measuring PWV [17]. The measurement of stiffness by ultrasound has the advantages of being significantly quicker and of not requiring a specific device.

Several studies, the largest of which was undertaken by Sutton-Tyrrell et al. and included 2488 patients, have used Doppler ultrasound to measure aortic PWV. Table 4 sets out the main PWV studies together with the methods employed [18]. However, few studies have compared a mechanical method with the use of Doppler [19].

**Table 4:** Studies with aortic PWV [18]

First author, year	Population (size)	Device used
Anderson, 2009 [20]	Non-diabetic population (n = 174)	Doppler flow

Blacher, 1999 [21]	ESRD (n = 241)	Doppler flow

Boutouyrie, 2002 [22]	Hypertension (n = 1,045)	Mechanotransducer (Complior)

Choi, 2007 [23]	Chest pain patients (n = 497)	Angiography (right Judkins catheter)

Cruickshank, 2002 [10]	Diabetes (n = 394)	Doppler flow

Laurent, 2001 [24]	Hypertension (n = 1,980)	Mechanotransducer (Complior)

Mattace-Raso, 2006 [25]	Community-based adults (n = 2,835)	Mechanotransducer (Complior)

Meaume, 2001 [26]	>70 years (n = 141)	Mechanotransducer (Complior)

Mitchell, 2010 [27]	General population (n = 2,232)	Arterial tonometry

Pannier, 2005 [28]	ESRD (n = 305)	Mechanotransducer (Complior)

Shoji, 2001 [29]	ESRD (n = 265)	PWV meter (PWV -200)

Shokawa, 2005 [30]	Ethnic minority (n = 492)	Mechanotransducer (MCG400)

Sutton-Tyrrell, 2005 [11]	Old adults (n = 2,488)	Doppler flow

Terai, 2008 [31]	Hypertension (n = 676)	Mechanotransducer (FCP-473)

Wang, 2010 [32]	General population (n = 1,272)	Arterial tonometry

Willum-Hansen, 2006 [33]	General population (n = 1,678)	Piezoelectric transducers (Hellige GmbH)

Zoungas, 2007 [34]	ESRD (n = 207)	Mechanotransducer (Millar Mikro-tip)

This study demonstrates that Doppler ultrasound can be used to measure aortic PWV in a reliable and reproducible way, giving similar results to Complior^®^, which we took as a gold-standard. In addition, B-mode ultrasound provides an anatomical image that can increase the precision of measurements (for example, using the carotid or femoral bifurcation as a reference). This method has the further advantages of shorter performance time, short learning curve and the absence of anatomical limitations, which are especially pronounced in the carotid artery. The versatility of ultrasound also permits us to explore simultaneously other pathologies such as plaques or blockages in the carotid and femoral territories as well as to assess intima-media thickness.

## Conclusions

The aim of this study was to consider the use of Doppler as an alternative to a more established method (Complior^®^) of measuring pulse wave velocity and to highlight certain advantages that Doppler has over this reference technique. Although the number of patients studied in this first study to specifically compare these two devices is limited, the findings are sufficiently powerful to demonstrate a high correlation between the two systems and so to justify the use of Doppler ultrasound both in clinical practice and clinical studies to assess arterial stiffness. However, automated methods should be developed for calculating transit time to reduce the variability from the use of manual calipers.

## Competing interests

The authors declare that they have no competing interests.

## Authors' contributions

JC designed the study, supervised subject recruitment, carried out collection of data and part of assessment of PWV by doppler and wrote the manuscript. PT carried out assessment of PWV by Complior, assisted in recruitment and manuscript revision. MG helped in the interpretation of the results and statistical analysis. IG assisted data interpretation and wrote the manuscript. NM and DF assisted recruitment, data collection and manuscript revision. BG assisted in assessment of PWV by both methods and participated in the analysis of reproducibility. MV assisted in study design, data interpretation and manuscript revision. All authors read and approved the manuscript.
